# Metal–Drug Complexes as Long‐Release Application for Antimalarial PfFNT‐Inhibitors

**DOI:** 10.1002/cmdc.70356

**Published:** 2026-07-01

**Authors:** Finn Tiedjens, Björn Henke, Ulrich Girreser, Regina Scherließ, Eric Beitz

**Affiliations:** ^1^ Department of Pharmaceutical and Medicinal Chemistry Kiel University Kiel Germany; ^2^ Department of Pharmaceutics and Biopharmaceutics Kiel University Kiel Germany; ^3^ Priority Research Area KiNSIS Kiel University Kiel Germany

**Keywords:** chemoprevention, inhibitor, malaria, metal–drug complex, PfFNT

## Abstract

Inhibition of the *Plasmodium falciparum* formate‐nitrite transporter (PfFNT) has emerged as a promising strategy for antimalarial drug development. PfFNT inhibitors display high potency against blood‐stage parasites and have shown efficacy in vivo. Here, we demonstrate activity against liver‐stage parasites of the front‐running compound, (Z)‐4,4,5,5,5‐pentafluoro‐3‐hydroxy‐1‐(pyridin‐3‐yl)pent‐2‐en‐1‐one (BH267m), highlighting the potential for chemopreventive applications. Consequently, we investigated the formation of metal complexes of PfFNT inhibitors as a strategy to generate long‐acting depot formulations enabling sustained drug release. Complexes with Mg^2+^, Ca^2+^, and Zn^2+^ were prepared via chelation through the pharmacophoric vinylogous acid moiety and characterized by spectroscopic methods. The resulting solids formed amorphous particles in the low micrometer range with metal‐dependent aqueous solubilities. In vitro release studies using a dialysis assay revealed sustained, near zero‐order ligand release, with zinc‐based complexes displaying particularly low solubility and slow release kinetics. Release rates varied by approximately one order of magnitude depending on metal ion and ligand structure. Simulated plasma concentrations suggested sustained exposure of one‐ to tenfold of the respective in vitro EC_50_ values within the current ligand portfolio. These findings indicate that coordination of vinylogous acid motifs with metal ions may provide a basis for developing long‐acting depot formulations of chelating anti‐infective agents.

## Graphical abstract

*P. falciparum* formate‐nitrite transporter (PfFNT) inhibitors have emerged as a novel antimalarial class with potent in vivo and liver‐stage activity. Low‐solubility metal–inhibitor complexes were prepared as potential long‐acting injectables. Selected complexes display sustained, near zero‐order ligand release, resulting in simulated plasma concentrations 1–10 × EC_50_.

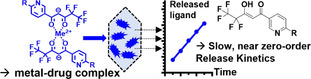

## 1 Introduction

Despite decades of progress, malaria remains a major global health burden, with approximately 282 million cases and 610,000 deaths reported in 2024, with recent gains in incidence reduction having plateaued [1]. Current control strategies rely heavily on short‐course oral artemisinin‐based combination therapies [2, 3] and chemoprevention regimens that require repeated monthly dosing, imposing substantial adherence and implementation challenges, particularly in vulnerable populations such as children and pregnant women [4]. Although recently recommended malaria vaccines represent important progress, their partial and relatively short‐lived protection renders drug‐based prevention and treatment indispensable [5]. At the same time, the emergence and spread of resistance across major antimalarial classes [6, 7] underscores the urgent need for agents with novel mechanisms of action and activity across multiple parasite stages [8]. In parallel, strategic priorities in the field have shifted toward long‐acting oral and injectable chemoprotective interventions [9, 10]. They are designed to provide durable exposure with infrequent dosing, thereby improving adherence, operational feasibility, and resilience against resistance‐driven failure of short‐acting regimens [4]. These trends emphasize the importance of combining novel target‐directed agents with long‐acting exposure profiles.

To this end, the identification of PfFNT, the l‐lactate transporter of *Plasmodium falciparum*, established a novel and mechanistically distinct antimalarial target [11]. This follows the rationale that blood‐stage parasites depend on a high glycolytic flux and continuous l‐lactate export for survival. Its essentiality for asexual parasite growth [12] and pharmacological tractability prompted target‐based screening efforts, including the Malaria Box, from which MMV007839 was identified as an initial hit with sub‐micromolar activity [13]. Subsequent medicinal chemistry optimization focused on improving potency and mitigating resistance liabilities, leading to the introduction of a pyridine core scaffold that overcame force‐selected mutations while maintaining activity against all five human‐pathogenic *Plasmodium* species [14]. The frontrunner compound BH267m **1** from this stage achieved 99.9% parasite clearance in vivo at 4 × 10 mg/kg [15]. However, in addition to efficacy against an active blood‐stage infection, a key criterion for chemopreventive use is a sufficient liver stage activity, as sporozoites infect the liver for 7 days after host infection [16]. Parasite eradication in this stage would prevent clinical disease as well as onward transmission [17]. This is taken into account in the respective Target Candidate Profile (TCP‐4) [18] of the Medicines for Malaria Venture (MMV), setting the goal for liver‐stage activity to match or exceed blood‐stage activity, which is investigated in this work for **1** (blood‐stage EC_50_ = 240 nM at 91% albumin binding; EC_50_free_comp._ = 21.6 nM).

Throughout this development, structure–activity studies revealed a conserved pentafluoro‐substituted vinylogous acid as the defining pharmacophore of the series. This motif likely mimics two consecutive lactate molecules in the PfFNT transport path, one in its protonated more lipophilic form, and one in its anionic form. The latter establishes a strong hydrogen donor/acceptor arrangement with His230 and Thr106, as supported by the ligand‐bound cryo‐EM structure [19]. Through the strong negative inductive effect mediated by the fluorine atoms, the acidity of the vinylogous acid (e.g., pK_a_ [calc.] of 4.82 for **1**) approximately matches that of lactic acid (pK_a_ 3.85 [20]) enabling pH‐dependent engagement of the transporter pathway. Later introduction of para‐substituents extending into the transporter vestibule further enhanced potency into the low‐nanomolar range but was accompanied by lowered metabolic stability [21]. Compound **1** as the currently metabolically most stable PfFNT inhibitor exhibits a microsomal clearance (human) of 3.98 ml/min/kg [21] and an in vivo half‐life (mouse) of 5.9 h [15], which is suitable for acute treatment, yet prevents its use as a chemopreventive.

We took this as an opportunity to seek modifications with the ability to extend the persistence of these compounds and investigate their potential application as long‐acting injectables (LAI). Although there is a variety of advanced options with different degrees of formulation technology available for LAI formulation [22], we aimed to explore directly compound‐related cost effective strategies, as these are advocated by the MMV Target Product Profiles [18].

Because the acidic functional group provides hydrophilicity and charge and is conserved in all relevant derivatives, it was the primary vantage point to create a pro‐drug form. We excluded a commonly employed alkylation or esterification strategy because attachment of these structural elements to the vinylogous acid moiety appeared too unstable and consequently are not described in the literature. Similarly, it is unlikely that capturing the enolate structure as silyl ethers [23] generates long‐term resistance against hydrolysis. We figured that complexation of the vinylogous acid in the tautomeric diketone form with divalent cations may provide sufficient stability depending on central ion [24, 25]. Metal‐containing compounds have long attracted interest in antimalarial drug development. Most prominently, the ferrocenyl chloroquine analog Ferroquine demonstrated potent activity against chloroquine‐resistant *P. falciparum* strains and advanced into clinical development, highlighting the therapeutic potential of organometallic antimalarials [26]. In these approaches, transition metals are typically incorporated as a direct pharmacophoric or redox‐active component. More recently, drug‐loaded metallic nanoparticles have been investigated for modulation of physicochemical and pharmacokinetic properties, including solubility, stability, and drug release behavior [27]. In contrast to classical metallodrugs, the present study employs biocompatible metal ions primarily as structural elements to generate low‐solubility depot complexes enabling sustained release of PfFNT inhibitors. This coordination chemistry based approach was applied and tested in this work.

## 2 Results and Discussion

### 2.1 Determination of Liver‐Stage Activity

Suitability of the inhibitor class towards the application as chemoprotective agent was validated through testing the front‐running compound **1** as a representative example of pyridine based PfFNT inhibitors in a liver stage maturation assay. This was conducted by incubating primary human hepatocytes and added sporozoites (Nijmegen *falciparum* (NF) 175 Nigeria strain) [28] with **1** for 4 days. Determining the percentage of infected hepatocytes by immunofluorescence staining revealed a remarkable EC_50_ of 55 ± 32 nM (Figure S6). This is within the same order of magnitude as to the blood‐stage EC_50_ of 21.6 nM for the free compound **1**. The atovaquone reference yielded an EC_50_ of 5.00 ± 5.41 nM. This comparable activity against both blood‐ and liver‐stage parasites represents an important characteristic for chemopreventive application, as inhibition of hepatic parasite maturation is a prerequisite for sterile protection and aligns with the MMV TCP‐4 profile [18]. In the context of long‐acting injectable formulations, sustained systemic exposure combined with potent liver‐stage activity may enable suppression of early parasite development following infection. These findings therefore confirm the potential of PfFNT inhibitors as multistage‐active antimalarial agents and strongly supports the feasibility for development as long‐acting chemopreventive agents.

### 2.2 Preparation of PfFNT Inhibitor–Metal Complexes and Spectroscopic Analysis

**SCHEME 1:**
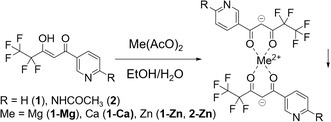
Complex formation from pentafluoro‐3‐hydroxy‐pent‐2‐en‐1‐ones with bivalent metal acetate salts (Mg, Ca, Zn).

Preparation was done by dissolving ligand **1** or **2** in ethanol at 60 °C, followed by addition of an aqueous metal acetate solution in one portion. After cooling to room temperature and addition of water, the resulting precipitate was filtered, washed with ethanol/water 1:1 and water, and dried in vacuo. This procedure afforded the herein described metal complexes in good to excellent yield (Scheme 1). In line with the straightforward synthesis of this PfFNT inhibitor class (one to three steps), metal complex formation represents a convenient strategy to introduce a controlled release mechanism without adding synthetic complexity or cost.

The choice of divalent metal ions was guided by biocompatibility considerations; thus, Mg^2+^ (**1‐Mg**), Ca^2+^ (**1‐Ca**), and Zn^2+^ (**1‐Zn, 2‐Zn**) were investigated. The proposed structure is based on related 1,3‐diketone metal complexes described in the literature [25]. After drying, additional coordination of H_2_O and ethanol is not observed for **1‐Mg** and **1‐Zn**, for **1‐Ca** coordinated ethanol remains.

**FIGURE 1:**
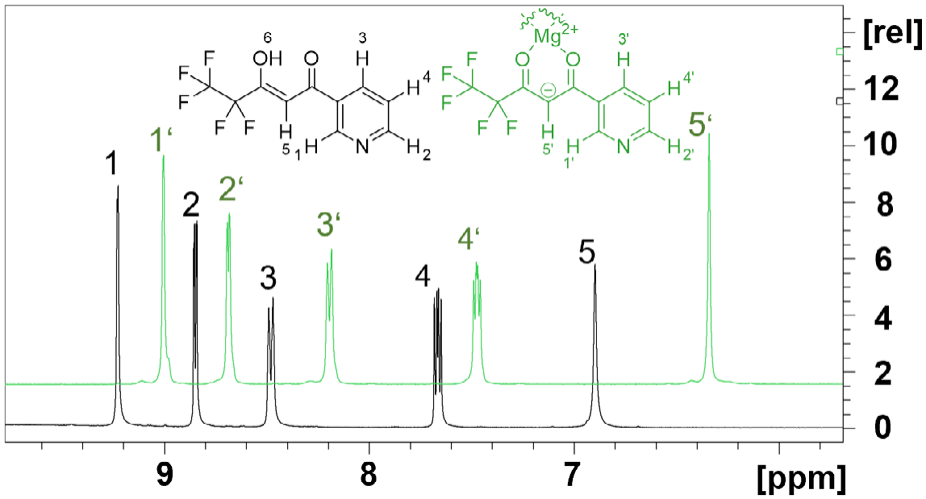
Overlay of 1H NMR spectra of **1** (black) and **1‐Mg** (green) with assigned protons.

^1^H NMR analysis of the DMSO‐*d_6_* soluble complexes of **1** (**1‐Mg** and **1‐Ca)** confirms the integrity of the ligand scaffold and is consistent with the published spectra of **1** [14]. Upon complexation, distinct upfield shifts were observed for the metal‐bound ligands (Figure 1). In agreement with the proposed deprotonation, the resulting negative charge within the vinylogous acid moiety increased electronic shielding. This effect was most pronounced at the methylene position H5, showing Δ*δ* = −0.557 ppm **1‐Mg** and −0.512 ppm **1‐Ca** (Table 1). The magnitude of the shift decreased toward the periphery of the molecule, reaching a minimum at H2 (4‐position of the vinylogous acid moiety) with Δ*δ* = −0.161 ppm **1‐Mg** and −0.139 ppm **1‐Ca**. Overall, the shifts were consistently smaller for the Ca^2+^ complex (**1‐Ca**). This can be attributed to its lower Lewis acidity due to the larger ionic radius compared to Mg^2+^ [29, 30], resulting in weaker polarization of the ligand framework and a less pronounced redistribution of electron density. Due to the even higher lewis acidity of Zn^2+^, the NMR shifts between **2** and **2‐Zn** are further increased with a maximum of −0.832 ppm for the methylene proton (Figure S4). NMR shifts between complex and ligand, most prominent in the vinylogous acid motif, are also observable in ^13^C and ^19^F spectra, further validating the described electron redistribution (Table S1).

**TABLE 1:** 1H NMR shifts induced by metal (Mg^2+^, Ca^2+^) complexation in ppm for **1‐Mg** and **1‐Ca** compared with free ligand **1**. DMSO‐*d6* used as solvent. Spectrum for **1‐Zn** could not be recorded due to poor solubility in standard deuterated solvents at room temperature and heated. Shifts between **2‐Zn** and **2** visualized in Figure S4. Proton 6 not observed. Integrals depend on number of complexed ligands. Spin–Spin coupling analogous to published NMR data of **1** [14].

Proton	*δ* 1	*δ* 1‐Mg	*δ* **1‐Ca** a	Δ*δ* 1 1‐Mg	Δ*δ* 1 1‐Ca
1	9.229	9.004	9.068	−0.224	−0.161
2	8.849	8.688	8.711	−0.161	−0.139
3	8.481	8.195	8.268	−0.286	−0.213
4	7.666	7.475	7.516	−0.192	−0.151
5	6.898	6.342	6.387	−0.557	−0.512

a 1H‐NMR spectrum for **1‐Ca** includes signals attributable to remaining ethanol as co‐ligand (1 equivalent, assuming a trimeric complex) not removable by additional drying (*δ* 1.0528 (t, 3H, CH_3_, ^3^ *J* = 7.0 Hz), *δ* 3.4391 (q, 2H, CH_2_, ^3^ *J*  = 7.0 Hz)).

**FIGURE 2:**
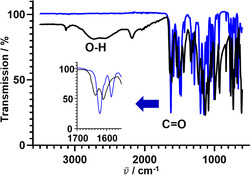
Overlay of ATR‐IR spectra of **1** (black) and **1‐Zn** (blue). Inset: $\bar{v}$ 1550–1700 cm^−1^ magnified.

To further probe the electronic effects of metal‐ligand binding and verify the coordination site, ATR‐IR spectra of compounds **1‐Mg**, **1‐Ca**, and **1‐Zn** were recorded (Figure 2, Table 2). Most notably, the broad O—H stretching signal (2180–3126 cm^−1^) observed for ligand **1** was significantly smaller in **1‐Ca** and absent in **1‐Mg** and **1‐Zn**. This observation is consistent with deprotonation and metal coordination via the 1,3‐dicarbonyl resonance structure. No additional O—H stretching bands attributable to coordinated solvent were observed in **1‐Mg** and **1‐Zn**, while **1‐Ca** exhibited signs of remaining ethanol ligands.

**TABLE 2:** ATR‐IR bands of compounds **1** and **1‐Mg**, **1‐Ca**, and **1‐Zn** for O—H and C=O stretch vibrations.

Vibration	[1] *T*/% ($\bar{v}$, cm^−1^)	[3] *T*/% ($\bar{v}$, cm^−1^)	[4] *T*/% ($\bar{v}$, cm^−1^)	[5] *T*/% ($\bar{v}$, cm^−1^)
O—H	82.01 br	—	89.78 br	—
	(2180–3126)		(2982–3096)^a^	
C=O	55.38 (1637)	39.20 (1627)	55.44 (1627)	25.02 (1622)
	48.47 (1610)	69.11 (1582)	76.07 (1571)	52.76 (1583)

a Assignable to ethanol as residual ligand.

The C=O stretching vibration (Figure 2, inset) appeared at 1622 cm^−1^ for **1‐Zn** and showed decreased transmission compared to the free ligand, consistent with an enhanced carbonyl character. The magnitude of this effect follows the order Ca^2+^ > Mg^2+^ > Zn^2+^, suggesting metal‐dependent modulation of electron density within the chelate system. This trend likely reflects differences in the ionic radius and Lewis acidity [29, 30].

**FIGURE 3:**
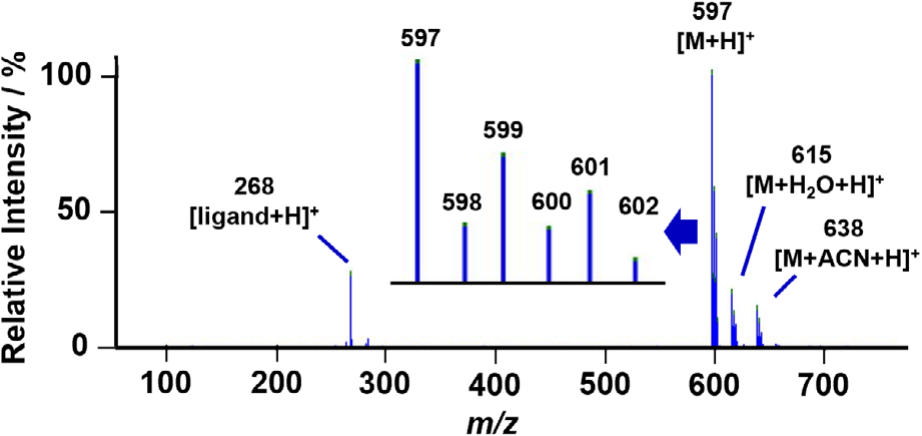
ASAP‐APCI + mass spectrum of **1‐Zn** with assigned peaks. Inset: [M + H]^+^ cluster, showcasing Zinc isotope pattern.

To test for the proposed dimeric complex structure, mass spectrometry using APCI was carried out. Due to complete dissociation under LC conditions resulting in exclusive detection of the free ligand, ASAP (Atmospheric Solids Analysis Probe [31]) was employed for sample introduction. This approach enabled detection of intact dimeric complexes for Mg^2+^ (m/z [M + H]^+^ 557 for **1‐Mg**, Table 3) and Zn^2+^ (m/z [M + H]^+^ 598 for **1‐Zn**, 711 for **2‐Zn**; Table 3, Figure 3).

**TABLE 3:** ASAP‐APCI + mass spectrometry with nominal resolution, AAS quantification, and solubility in PBS pH 7.4 (37  °C) for metal complexes **1‐Mg**, **1‐Ca**, **1‐Zn**, and **Zn‐2**. Relative intensity related to highest peak in the isotope cluster. **1‐Ca** not found in mass analysis (n.d. – not determined).

	1‐Mg	1‐Ca	1‐Zn	2‐Zn
Exact *m/z* calculated for [M + H]^+^ (relative intensity, %)	557 (100) 558 (21.9) 559 (14.2)	573 (100) 574 (22.5) 575 (2.3) (840 as three–ligand complex)	597 (100) 598 (21.9) 599 (59.6) 600 (20.8) 601 (41.3) 602 (8.7)	711 (100) 712 (27.6) 713 (61.3) 714 (23.2) 715 (42.6) 716 (10.5)
*m/z* found for [M + H]^+^ (relative intensity, %)	557 (100) 558 (32.5) 559 (18.1)	n.d.	597 (100) 598 (26.4) 599 (59.1) 600 (24.5) 601 (40.9) 602 (10.0)	711 (100) 712 (25.8) 713 (57.3) 714 (25.8) 715 (35.5) 716 (16.0)
Further *m/z* found for M [adduct]^+^	575 [M + H_2_O + H]^+^	n.d.	615 [M + H_2_O + H]^+^ 638 [M + ACN + H]^+^	n.d.
Calculated metal content, %	4.37	7.00 (4.77 as three–ligand complex)	10.94	n.d.
Found metal content, %	4.32 ± 0.04	4.87 ± 0.02	10.91 ± 0.06	n.d.
Solubility, mg/L	173	247 (241 as three–ligand complex)	24	n.d.

AAS analysis revealed metal contents of 4.32 ± 0.04% (m/m) **1‐Mg** and 10.91 ± 0.06 % (m/m) **1‐Zn**, corresponding to recoveries of 98.9% and 99.7%, respectively, for the proposed dimeric structures (Table 3). The Ca^2+^ complex **1‐Ca** was not sufficiently stable for detection by ASAP‐APCI mass spectrometry. Notably, its metal content determined by AAS suggests a trimeric complex (recovery 102.1%) rather than the dimeric motif observed for Mg^2+^ and Zn^2+^. This divergent behavior may be attributed to the comparatively large ionic radius of Ca^2+^ [29], potentially enabling coordination of a third ligand. Due to poor crystallization behavior of the compounds, the achieved crystalloids in a screening of several solvents proved insufficient for single‐crystal X‐ray analysis; therefore, corresponding data is omitted.

### 2.3 Characterization of Precipitated Metal Complex Particles

As the goal of this study was to functionally characterize ligand release from the complexes, we next investigated their particle properties. As described by the Noyes–Whitney equation [32], the dissolution rate of particles is proportional to their surface area. We therefore analyzed particle size as a key parameter. Laser diffraction analysis showed similar *x*_50_ values of 10.37 and 12.42 µm for the Mg^2+^ and Ca^2+^ complexes **1‐Mg** and **1‐Ca**, respectively, whereas the analogous Zn^2+^ complex **1‐Zn** yielded approximately one order of magnitude smaller particles with an *x*_50_ of 1.52 µm (Table 4). This observed property aligns with the more rapid precipitation of the Zn^2+^ complex during preparation. The alternative Sauter mean diameter (SMD) weights the particle diameter to the surface‐to‐volume ratio and is therefore particularly relevant for dissolution studies. Here, the differences between the complexes decreased somewhat. Still, the SMD for **1‐Zn** (1.04 µm) remained significantly smaller than for **1‐Mg** (3.00 µm) and **1‐Ca** (4.98 µm). Switching the ligand to **2** yielded Zn^2+^‐complex particles **2‐Zn** of *x*_50_ (1.63 µm) and SMD (1.05 µm) that closely matched the particle sizes of **1‐Zn** indicating a minimal influence of ligand substitution on this parameter.

**TABLE 4:** Particle size characterization by laser diffraction for metal complexes **1‐Mg**, **1‐Ca**, **1‐Zn**, and **2‐Zn** stated as percentile diameters, volume mean diameter (VMD), and *Sauter* mean diameter (SMD). Values stated as Mean ± SD (*n* = 3).

	1‐Mg	1‐Ca	1‐Zn	2‐Zn
*X*_10_, µm	0.92 ± 0.14	1.83 ± 0.26	0.49 ± 0.01	0.48 ± 0.03
*X*_50_, µm	10.37 ± 1.44	12.42 ± 0.14	1.52 ± 0.02	1.63 ± 0.25
*X*_90_, µm	20.61 ± 0.43	25.79 ± 1.13	4.10 ± 0.06	4.52 ± 0.19
SMD, µm	3.00 ± 0.60	4.98 ± 0.28	1.04 ± 0.02	1.05 ± 0.08
VMD, µm	10.42 ± 1.03	13.46 ± 0.22	1.98 ± 0.01	2.20 ± 0.16

**FIGURE 4:**
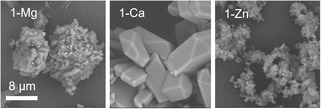
SEM images of metal complexes **1‐Mg**, **1‐Ca**, and **1‐Zn** at ×10,000 magnification.

Visualization of the particles from complexes **1‐Mg**, **1‐Ca** and **1‐Zn** by scanning electron microscopy (SEM) confirmed the laser diffraction data and revealed morphological differences. Here, **1‐Mg** and **1‐Ca** were similar in size, with a more amorphous‐like morphology of the Mg^2+^ complex **1‐Mg** and a crystalline appearance of the Ca^2+^ complex **1‐Ca**. The Zn^2+^‐based complex **1‐Zn** produced mainly fine‐grained amorphous particles (Figure 4). In terms of surface‐to‐volume ratio, the particles can be ranked Zn^2+^ > Mg^2+^ > Ca^2+^.

### 2.4 Kinetics of Inhibitor Release From Metal Complexes

**FIGURE 5:**
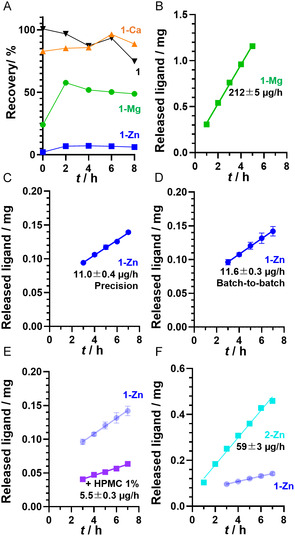
Release‐kinetics of metal complexes. (A) Recovery of ligand under intense mixing of suspension in PBS pH 7.4, 37 °C, non‐sink conditions. (B–F) Released ligand from suspension (stabilizer Tween80 0.1%) in dialysis tube under stirring in PBS pH 7.4, 37 °C, sink conditions. Assessing influence of changing metal ions (B and C), ligand (F), and suspension viscosity (HPMC4000, 0.5%) (E). Internal validation of precision (technical replicates) and batch‐to‐batch reproducibility with error bars denoting standard deviation, *n* = 3. Y‐offset normalized (C and D).

To assess the suitability of the complexes for application as a slow‐release formulation, an initial solubility experiment was done. Complexed compounds **1‐Mg**, **1‐Ca**, and **1‐Zn** in amounts equaling 10 mM of ligand **1** and free compound **1** as a standard were suspended in saline buffer (pH 7.4) under vigorous shaking under non‐sink conditions (Figure 5A). Release profiling revealed that **1‐Ca** immediately freed 70% of its ligand during the setup of the experiment and 100% over the following 6 h accompanied by the visible dissolution of the suspended particles. This is in stark contrast to **1‐Mg** and **1‐Zn**, which reached a solubility cap and only released approximately 50% and 7%, respectively, of their ligand. Quantified after 2 h incubation, the solubility of the complexes corresponds to 173 mg/L for **1‐Mg**, 241 mg/L for **1‐Ca** and 24 mg/L for **1‐Zn** (Table 3). The slight decrease in the concentration of **1** is caused by hydrolysis of the vinylogues acid in a Retro–Claisen condensation (*t*_1/2_ 24 h), resulting in hydrophilic, non‐active products. As a result of these findings, the Ca^2+^‐complex **1‐Ca** was excluded from further investigations as it proved to be too unstable in aqueous media.

For determining the release behavior under sink conditions, the metal complexes were placed in dialysis tubes as 10.0 ± 0.1 mg suspensions in saline buffer (pH 7.4) containing 0.1% Tween80 as a stabilizing agent. The surrounding buffer was stirred, periodically sampled for released ligand, and regularly replaced to maintain sink conditions. This method is widely used for in vitro release studies of LAIs [33] and allows for estimations of in vivo drug release rates. A potential limitation of this method is retention of the ligand by the dialysis membrane and thus distorted release profiles [34]. We excluded this issue by monitoring the release of free ligand **1**, which showed a satisfactory faster rate than the metal–ligand complexes (Figure S7). Compounds **1‐Mg** and **1‐Zn** (Figure 5B,C) showed a linear increase in the concentration of released free ligand **1** over 7 h (*r* ^2^ > 0.99). A zero‐order release profile represents an ideal prerequisite for developing a long‐acting drug formulation that builds a constant plasma concentration in the therapeutic window while minimizing dosing frequencies [35]. The Mg^2+^‐based complex **1‐Mg** showed a release‐rate of 212 ± 5 µg/h. Zn^2+^‐based complex **1‐Zn** exhibited a more than one order of magnitude slower release of 11.0 ± 0.4 µg/h. In view of the much smaller particle size and SMD this indicates substantially stronger ligand binding of the Zn^2+^‐complexes. We further validated the method using **1‐Zn** for technical reproducibility (Figure 5C; *n* = 3, slopes not significantly different, ANCOVA, *p* = 0.4804) and batch‐to‐batch reproducibility (Figure 5D; *n* = 3, slopes not significantly different, ANCOVA, *p* = 0.6522), with a finalized release rate of 11.6 ± 0.3 µg/h.

Next, we tested how changes in the size and composition of the depot affects ligand release. First, we decreased the depot for compounds **1‐Zn** and **2‐Zn** to 1 mg resulting in an approximately twofold reduction in the release rate (e.g., **Zn‐1** with 5.4 ± 0.3 µg/h; Figure S8). This comparatively small change in the release rate despite a tenfold reduction in depot size indicates a high stability in release over time, even in a shrinking depot. Additionally, for **1‐Zn,** release rates for 5 and 50 mg (9.1 ± 0.5 and 7.7 µg/h ± 0.5 respective; Figure S9) were determined. While the first supports the notion of a slow decrease in release rate from a 10 mg depot, the latter shows a slower release rate likely due to more pronounced particle agglomeration inside the dialysis tube. While longer‐term release experiments were not conducted due to the inherent hydrolytic instability of the molecules, this data allows the assumption of near zero‐order release over a large fraction of depot disintegration. Second, as a proof‐of‐concept, we probed the option of influencing the release rate with excipients by increasing the viscosity through addition of 0.5% hydroxypropyl methylcellulose (HPMC) 4000–10 mg depots of **Zn‐1**. This decreased the release rate twofold (5.5 ± 0.3 µg/h) due to slowed diffusion out of the reservoir (Figure 5E). Eventually, we prepared complex **2‐Zn** as an alternative to **1‐Zn**, keeping Zn^2+^ as the central cation, but changing the ligand to **2** (Scheme 1). The choice of ligand was made upon superior in vitro activity with an EC_50_ of 49.8 nM (3 nM free compound at 94% albumin binding), yet at the cost of decreased metabolic stability (Cl_int_ of 10 mL/min/kg [21]). For a 10 mg depot of **2‐Zn** in saline/Tween80 buffer, a release rate of 59 ± 3 µg/h was determined, i.e., approximately six times faster than **1‐Zn** (Figure 5F). This can be attributed to a hydrolysis‐promoting destabilization of the ligand anion, which is mediated by a positive mesomeric effect of the acetanilide group. Following this finding, complexes with a slower release‐profile, even with Mg^2+^ and Ca^2+^ as central ions, could be synthesized using a ligand substituted with electron‐withdrawing groups, thus stabilizing the coordination bond. However, the choice of ligand would be mainly driven by pharmacodynamic and pharmacokinetic properties, favoring mostly electron‐donating substituents [21].

Assuming a theoretical depot mass of 100 mg releasing ligand at a constant rate (saline/Tween80 values), depots of **1‐Mg**, **1‐Zn** and **2‐Zn** would last 19, 359, and 70 days, respectively. These numbers are estimations lacking experimental validation for such time spans and only serve to assess and differentiate the drug‐retaining ability of the compounds. In this context, they show the potential of Zn^2+^‐based PfFNT inhibitor complexes for reaching the goal for an LAI release duration of 1–6 months as defined in the MMV Target Product Profile. Although in vivo data is ultimately needed to assess in vitro‐in vivo correlation (IVIVC), is has been shown for LAIs that good IVIVC can be achieved with the use of the dialysis‐bag method [36]. Upon i.m./s.c. administration, the injectible would be immobilized and dissolved slowly into a partly stagnant fluid layer before getting exposed to the sink‐conditions of the surrounding tissue. This release process is adequately approximated by the here described method, with the adjusted pH of 7.4 matching the interstitial pH in muscle tissue [37]. It is to be noted that the dissolution time in vivo is expected to vary with the amount of blood flow as well as the acidity at the chosen injection site. Due to the anionic form of the complexed inhibitor, the dissociation rate is expected to increase with rising tissue acidity.

### 2.5 Simulation of Plasma Levels

Finally, the putative plasma concentrations were simulated over a time period of 28 days based on measured release rates and pharmacokinetic data for complexes **1‐Zn** and **2‐Zn** and their respective ligands **1** and **2** plus the following assumptions. The release kinetics were assumed to follow zero‐order behavior based on the obtained short‐term data (Figure 5D,F) and the small decrease in release rate resulting from changing depot size. Due to the previously determined high plasma protein binding PPB (**1**, 91%; **2**, 94% [21]) and very low distribution volume *V*_d_ (**1**, 0.3 L/kg, in vivo mouse; **2** expected equal based on logD7.4 and PBB), rapid distribution from the injection site into the blood with limited tissue penetration was being assumed [38]. Human in vivo clearances were predicted from in vitro human microsomal clearance and physicochemical data [21] (**1**: 3.98 → 0.032 mL/min/kg; **2**: 10.64 → 0.059 mL/min/kg) using the MMV Sola tool [39].

**FIGURE 6:**
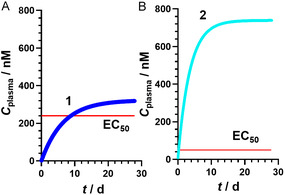
Simulated plasma concentrations of inhibitors **1** and **2** released linearly from metal complexes **1‐Zn** (A) and **2‐Zn** (B). *CL*_human_ was estimated from *CL*_human_ _microsomal_ using the MMVSola software.

Under these conditions, the simulation of complex **1‐Zn** showed a very slow build‐up of ligand due to a low release rate (Figure 6A). A 10‐day time period may be required until the plasma concentration level corresponding to the in vitro EC_50_ of 240 nM (albumin bound) is reached, and a maximal plasma concentration of 317 nM can be expected. Due to high risk of drug‐resistance formation [12] and expected low efficacy in this concentration range this release profile is likely not sufficient, if not given in combination. Compound **2‐Zn** carrying the more potent ligand **2** in turn may exceed the in vitro EC_50_ of 49.8 nm (albumin bound) within 6 h after application due to the six times higher release rate in combination with an only 2‐fold increased *CL*_human_ of ligand [2] (Figure 6B). Importantly, a steady‐state plateau indicating a maximal plasma concentration of 739 nM (>10‐fold EC_50_) is predicted. Although the simulation data must be interpreted with caution, this shows that further investigations of **2‐Zn** (and similar Zn^2+^ complexes of ligands with optimized PK and PD profiles) for their chemopreventive potential is a worthwhile endeavor.

## 3 Conclusion

In this study, we demonstrated the potential of PfFNT inhibitors applied as chemopreventive agents through the finding of high liver‐stage activity for the representative frontrunning compound. Building on this groundwork, we describe the synthesis of divalent metal complexes of PfFNT inhibitors through chelation via their pharmacophoric vinylogous acid moiety. The resulting complexes form predominantly amorphous microparticles with metal‐dependent aqueous solubilities and particle sizes. Among the investigated biocompatible metals, zinc‐based complexes displayed particularly low aqueous solubility and enabled sustained ligand release with near zero‐order kinetics. Simulated plasma concentrations estimated from these release rates and previously obtained pharmacokinetic properties yielded lasting steady‐state concentrations up to tenfold higher than the in vitro EC_50_. This highlights the potential of this approach for sustained delivery of PfFNT inhibitors. Overall, these findings establish metal coordination of vinylogous acid motifs as a promising strategy for generating long‐acting depot formulations of small‐molecule anti‐infectives and provide a foundation for future optimization of ligand structures and formulation parameters.

## 4 Experimental Section

### 4.1 Liver Stage Maturation Assay

Cryopreserved primary human hepatocytes were thawed and seeded at 18,000 cells per well in a collagen coated 384 well plate. Three hours post‐thawing, all non‐adhered cells were removed, and the adhered cells were incubated for 2 days. Salivary gland sporozoites were isolated from mosquitoes infected with NF175 P. falciparum. For each well, 15,000 sporozoites were transferred onto the hepatocytes and after 3 h, non‐invaded sporozoites were aspirated and the cells were incubated for 4 days in the presence of compound. To this end, compound was dissolved in DMSO to a concentration of 10 mM. Subsequently, 1 was serially diluted in DMSO and then in hepatocyte growth medium to achieve a final DMSO concentration of 0.1%. Half of the medium was refreshed daily with fresh compound. Hepatocytes were fixed and stained with anti‐PfHSP70 and DAPI. The number of hepatocyte nuclei and PfHSP70 positive forms were quantified by automated imaging. Three independent experiments with two technical replicates were conducted. Data was expressed as % inhibition relative to vehicle control on assay plate. Quality criteria of >20 infected primary hepatocytes per well treated with vehicle and an EC_50_ of atovaquone (positive control) meeting the historical average of 8 ± 24 nM had to be met. Data was fitted through four‐parameter nonlinear regression model, using the least squares method to find the best fit. Reported error for EC_50_ denotes SD. Experiments were conducted and evaluated by Youri van Nuland and Robert Sauerwein (TropIQ Helath Sciences, Nijmegen, The Netherlands).

### 4.2 Preparation of Complexes

Ligand (1.02 mmol of **1** [15] for complexes **1‐Mg**, **1‐Ca** and **1‐Zn**, 0.116 mmol of **2** [21] for complex **2‐Zn**) was dissolved in ethanol_abs_ (5 mL for **1‐Mg**, **1‐Ca** and **1‐Zn**, 0.57 mL for **2‐Zn**)_._ at 60 °C. Solution of metal acetate (**1‐Mg** Mg(OAc)_2_.·4H_2_O (0.5 mmol), **1‐Ca** Ca(OAc)_2_.·1H_2_O (0.5 mmol), **1‐Zn** Zn(OAc)_2_.·2H_2_O (0.5 mmol), **2‐Zn** Zn(OAc)_2_.·2H_2_O (0.057 mmol)) dissolved in water (5 mL for **1‐Mg**, **1‐Ca** and **1‐Zn**, 0.57 mL for complex **2‐Zn**) was added in one portion. After cooling to room temperature, water was added (15 mL for complexes **1‐Mg**, **1‐Ca** and **1‐Zn**, 1.7 mL for **2‐Zn**). The resulting precipitate was filtered (for **1‐Mg**, **1‐Ca**) or centrifuged (for **1‐Zn** and **2‐Zn**) and washed with ethanol/water 1:1 and water. The solids were dried to mass constancy in vacuum at 60 °C to yield the title compounds:

**1‐Mg** 216 mg, 78%, ^1^H NMR (400 MHz, DMSO‐*d6*): δ/ppm 9.00 (s, 2H), 8.69 (d, ^3^ *J* = 3.6 Hz, 2H), 8.19 (d, ^3^ *J* = 8.0 Hz, 2H), 7.47 (dd, ^3^ *J* = 4.8 Hz, ^3^ *J* = 8.0 Hz, 3H), 6.34 (s, 3H). ^13^C NMR (101 MHz, DMSO‐*d6*): δ/ppm 186.0 (2C), 172.9 (2C), 152.3 (2C), 148.3 (2C), 134.7 (2C), 133.8 (2C), 123.7 (2C), 120.0 (2C), 117.1 (2C), 91.1 (2C). ^19^F (377 MHz, DMSO‐*d6*): δ/ppm −81.6 (6F), −121.5 (4F). MS (ASAP‐APCI positive) *m/z* (%): 557 (100) [M+H]^+^. AAS recovery as two‐ligand salt: 98.9%.

**2‐Ca** 230 mg, 55%, 1H NMR (400 MHz, DMSO‐d6): δ/ppm 9.07 (d, 4J = 2.0 Hz, 3H), 8.71 (dd, 4J = 1.5 Hz, 3J = 4.8 Hz, 3H), 8.27 (dt, 4J = 1.5 Hz, 4J = 2.0 Hz, 3J = 8.0 Hz, 3H), 7.52 (dd, 3J = 4.8 Hz, 3J = 8.0 Hz, 3H), 6.39 (s, 3H), ethanol co‐ligand: 3.44 (q, 3J = 7.0 Hz, 2H, CH_2_), 1.05 (t, 3J = 7.0 Hz, 3H, CH_3_). 13C NMR (101 MHz, DMSO‐d6): δ/ppm 183.5 (3C), 171.9 (3C), 151.7 (3C), 148.1 (3C), 135.2 (3C), 133.7 (3C), 123.7 (3C), 120.0 (3C), 117.1 (3C), 91.4 (3C), ethanol co‐ligand: 56.0 (1C, CH2), 18.5 (1C, CH3). 19F (377 MHz, DMSO‐d6): δ/ppm −81.6 (9F), −121.3 (6F). Compound not observed in MS due to low stability. AAS recovery as three‐ligand salt: 102.1%.

**1‐Zn** 252 mg, 85%, NMR data not obtainable due to poor solubility. MS (ASAP‐APCI positive) m/z (%): 597 (100) [M + H]+, AAS recovery two‐ligand salt: 99.7%.

**2‐Zn** 33 mg, 83%, ^1^H NMR (400 MHz, DMSO‐*d6*): δ/ppm 10.80 (s, 2H), 9.09 (s, 2H), 8.19 (dd, ^4^ *J* = 1.9 Hz, ^3^ *J* = 8.8 Hz, 2H), 8.10 (d, ^3^ *J* = 8.8, 2H), 6.25 (s, 2H), 2.11 (s, 6H). ^13^C NMR (101 MHz, DMSO‐*d6*): δ/ppm 186.1 (2C), 171.9 (2C), 169.7 (2C), 154.3 (2C), 147.7 (2C), 137.1 (2C), 129.3 (2C), 117.1 (2C), 112.2 (2C), 108.6 (2C), 90.4 (2C), 24.0 (2C). ^19^F (377 MHz, DMSO‐*d6*): δ/ppm −81.6 (6F), −121.1 (4F). MS (ASAP‐APCI positive) *m/z* (%): 711 (100) [M+H]^+^).

Further compound characterization is described above and in Supporting Information.

### 4.3 Chemical Analysis

NMR: NMR‐spectra were recorded on Avance III 400 NMR spectrometer (Bruker) at a frequency of 400 MHz (^1^H), 101 MHz (^13^C) or 377 MHz (^19^F) and temperature of 25 °C. Processing, calibration of spectra to solvent signal (DMSO‐*d6*, 2.50 ppm (^1^H), 39.51 (^13^C)), referencing to external standard (^19^F: CFCl3, 0.000 ppm), analysis and visualization was performed with TopSpin 3.6.1.

ATR‐IR: ATR‐IR‐spectra were recorded on Spectrum 100 ATR‐FT‐IR spectrometer (PerkinElmer) at 25 °C. Data visualization was performed with GraphPadPrism 11.0.0.

ASAP‐APCI mass spectrometry: Low resolution mass spectra were recorded in positive mode on a CMS Expression mass spectrometer (Advion) using chemical ionization at atmospheric pressure (APCI). Samples were introduced using atmospheric solids analysis probe (ASAP) for direct insertion into ionization chamber. Data analysis and visualization were performed with Advion Data Express 6.9.43.1.

Atomic absorption spectroscopy (AAS): Measurements were performed on a 3030 Atomic Absorption Spectrometer (PerkinElmer) using air‐acetylene oxidizing flame. Hollow‐cathode‐lamps at the following wavelengths were used: Mg (285.2 nm), Ca (422.7 nm), Zn 213.9 nm). Commercial calibration standards (Sigma–Aldrich) for 1000 mg/L were diluted to obtain the following linear calibration ranges: Mg (0.25–1.00 mg/L), Ca (1.25–5.01 mg/L), and Zn (0.50–1.96 mg/L). Samples were dissolved in 1% aqueous nitric acid and diluted with water to fall within the linear range. Metal concentration in mg/L was related to sample weight. Measurements were performed in triplicates, *n* = 3, and values are reported as mean ± SD.

### 4.4 Particle Characterization

Particle size distribution: Particle size distributions were measured by laser diffraction using a HELOS instrument equipped with a RODOS dry dispersion unit and R3 (for **1‐Mg** and **1‐Ca**) or R1 (for **1‐Zn** and **2‐Zn**) optical lens (instrument and modules manufactured by Sympatec GmbH). Samples were dispersed at 4 bar and analyzed using the Fraunhofer optical model. Measurements were performed in triplicates, *n* = 3, and values are reported as mean ± SD. Analysis and visualization were performed with PAQXOS 5.2.1.

SEM: Visualization by scanning electron microscopy was performed using a Phenom Pro XL instrument (Phenom‐World BV) and the compatible Phenom ProSuite Desktop SEM software. Samples were prepared by adhering to aluminum stubs using carbon stickers (Plano GmbH) and gold sputtering using a BAL‐Tec SCP 050 Sputter Coater (Bal‐Tec AG, Liechtenstein). Shown images were recorded at 10,000 × magnification and 10 kV accelerating voltage.

### 4.5 Determination of Solubility and Release Rate

Solubility: **1** (0.25 µmol) and **1‐Mg**, **1‐Ca** and **1‐Zn** (amounts corresponding to 0.25 µmol of complexed ligand) were suspended in 25 ml PBS pH 7.4. The tubes were incubated at 37 °C under vigorous shaking. Aliquots were taken at 2 h intervals, centrifuged, and the concentration of ligand **1** in the supernatant was quantified by UV‐Vis‐spectroscopy. Recovery of ligand was plotted using GraphPadPrism 11.0.0. Solubility as stated in Table 3 was calculated from ligand concentration after 2 h incubation.

Ligand release: Complex samples were weighed as described above and placed in reaction tubes (2 mL). Samples were suspended in 1.00 mL PBS (pH 7.4) containing 0.1% Tween 80 and vortexed until homogeneous. Suspensions were transferred into dialysis bags (length 3 cm, molecular weight cut‐off 14 kDa) and immersed in 29.0 mL PBS (pH 7.4). The vessels were stirred at 500 rpm and incubated at 37 °C. Aliquots of the external medium were taken at 1 h intervals, and the released ligand (**1** or **2**) was quantified by UV‐Vis spectroscopy. When necessary, the medium was replaced to maintain sink conditions. Release rates were determined by fitting for linear regression or by fitting an exponential plateau model (membrane diffusion control of **1**). Release rate determination was validated for complex **1‐Zn** using technical and batch‐to‐batch replicates (*n* = 3). Statistical comparison of slopes was performed by ANCOVA. Data analysis and visualization were performed using GraphPad Prism 11.0.0.

Quantification of ligand: For each ligand, the absorption maximum (**1**: 325 nm **2**: 332 nm), linear absorbance range (**1** 0.0783–0.7738, **2** 0.0980–0.8445) and calibration function (**1** *A* = 0.0156*x* + 0.0045, *r* ^2^ > 0.99; **2** *A* = 0.0232*x* + 0.0567, *r* ^2^ > 0.99; *A* absorbance, *x* concentration in µM) were determined. Samples were diluted to fall within the linear range, and absorbance was measured at the respective wavelength using PBS pH7.4 as blank.

### 4.6 Simulation of Plasma Concentration

Zero‐order ligand release combined with first‐order elimination was modeled using the following equation:

$$c=\frac{R}{Cl\cdot \mathrm{BW}\cdot 0.06}\cdot \left(1-e^{-\frac{CL\cdot \mathrm{BW}\cdot 0.06)}{V_{d}\cdot \mathrm{BW}}\cdot t}\right)\cdot \frac{10^{6}}{\mathrm{MW}}$$

where *c* is the concentration/nM, *R* the release rate/µg/h, *CL* the in vivo Clearance calculated from *CL*_human_ _microsomal_ [21] for ligands **1** 0.032 and **2** 0.059 mL/min/kg using MMV Sola [36], BW the body weight (assumed 70 kg), *V*_d_ = distribution volume in vivo mouse (measured for **1** as 0.3 L/kg [14], same value assumed for **2** as PPB and logD_7.4_ closely align with **1**), and MW the molecular weight.

Time was simulated over a period of 28 days, corresponding to the once‐monthly dosing target defined by MMV for chemoprevention [18]. Under the assumed release rates, this duration can be achieved using an exemplary depot size of 100 mg. Data simulation and visualization were performed using GraphPad Prism 11.0.0.

## Funding

This study was supported by the Deutsche Forschungsgemeinschaft (Be2253/8).

## Conflicts of Interest

The authors declare no conflicts of interest.

## Supporting information

Supplementary Material

## Data Availability

The data that supports the findings of this study are available in the supplementary material of this article.
