# Supplementary material for: Metal–Drug Complexes as Long‐Release Application for Antimalarial PfFNT‐Inhibitors
Source: ChemMedChem. 2026 Jul 1;21(13):e70356. doi: 10.1002/cmdc.70356 (PMC13321776; doi:10.1002/cmdc.70356)
Supplement: Supplementary file 1 — Supplementary Material [file CMDC-21-e70356-s001.pdf]

Supporting Information

Metal-Drug Complexes as Long-Release Application for Antimalarial PfFNT-Inhibitors

Finn Tiedjens, Björn Henke, Ulrich Girreser, Regina Scherließ and Eric Beitz

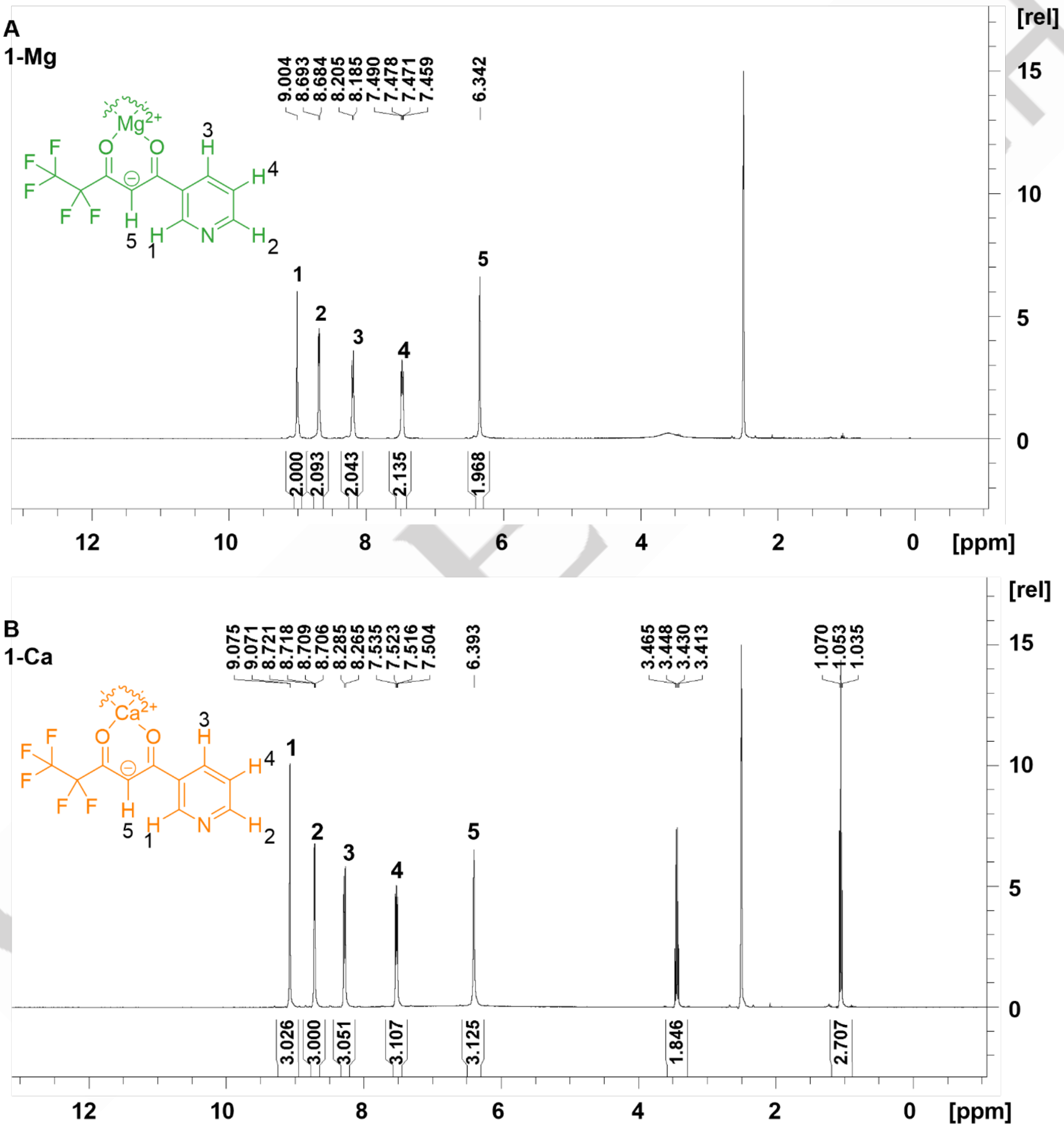

Figure S1. ^{1}H NMR spectra of **1-Mg (A)** and B **1-Ca (B)** (400 MHz, DMSO-*d6*).

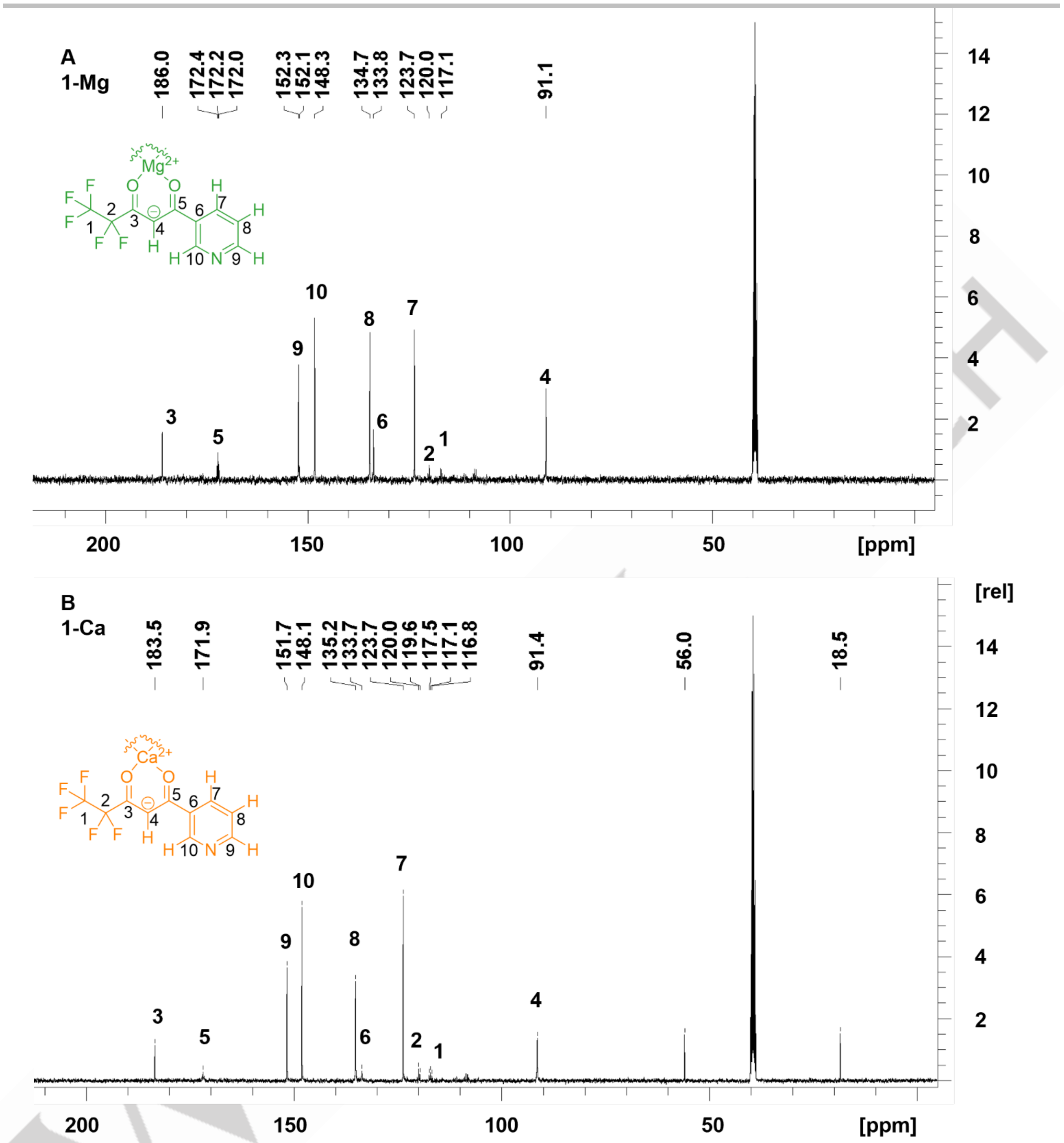

Figure S2. ^{13}C NMR spectra of **1-Mg (A)** and **1-Ca (B)** (101 MHz, DMSO-*d6*).

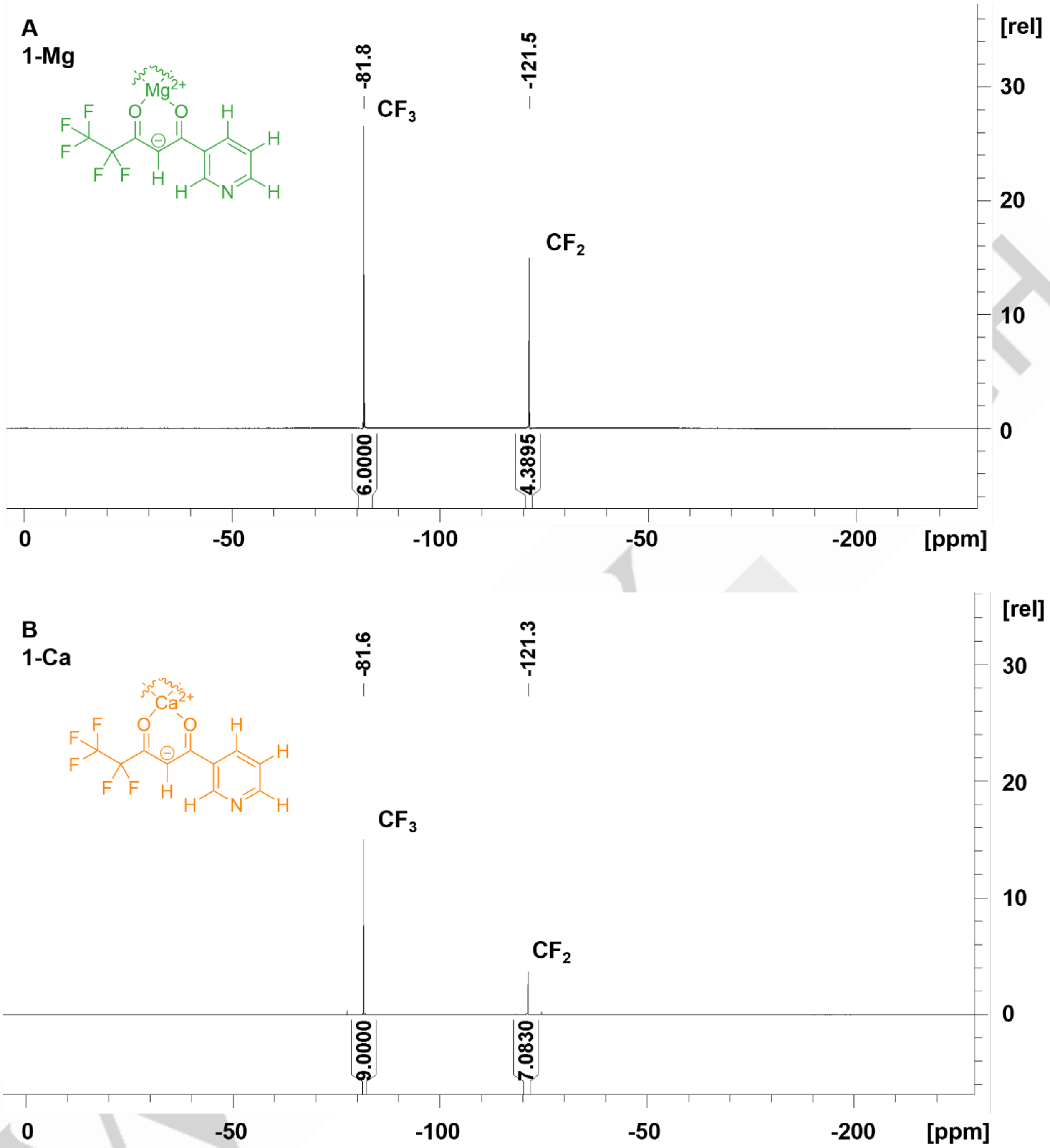

Figure S3. ^{19}F NMR spectra of **1-Mg (A)** and **1-Ca (B)** (377 MHz, DMSO-*d6*).

Table S1. 13C-NMR and 19F-NMR shifts induced by metal (Mg^{2+}, Ca^{2+}) complexation in ppm for **1-Mg** and **1-Ca** compared to free ligand **1**[14]. DMSO-*d6* used as solvent. Spectra for **1-Zn** could not be recorded due to poor solubility.

C/F	*δ* **1**	*δ* **1-Mg**	*δ* **1-Ca**[a]	*Δδ* **1 1-Mg**	*Δδ* **1 1-Ca**
1	116.6	117.1	117.1	0.5	0.5
2	119.5	120.0	120.0	0.5	0.5
3	182.9	186.0	183.5	3.1	0.6
4	94.4	91.1	91.4	-3.3	-3.0
5	174.5	172.2	171.9	-2.3	-2.6
6	130.3	133.8	133.7	3.5	3.4
7	124.3	123.7	123.7	-0.6	-0.6
8	136.6	134.7	135.2	-1.9	-1.4
9	152.6	152.3	151.7	-0.3	-0.9
10	148.0	148.3	148.1	0.3	0.1
CF_3	-81.8	-81.8	-81.6	0.0	0.2
CF_2	-122.3	-121.5	-121.3	0.8	1.0

[a] 13C-NMR spectrum for **1-Ca** includes signals attributable to remaining Ethanol as co-ligand ((δ 18.5 (1C, CH_3), δ 56.0 (1C, CH_2)).

WILEY-VCH

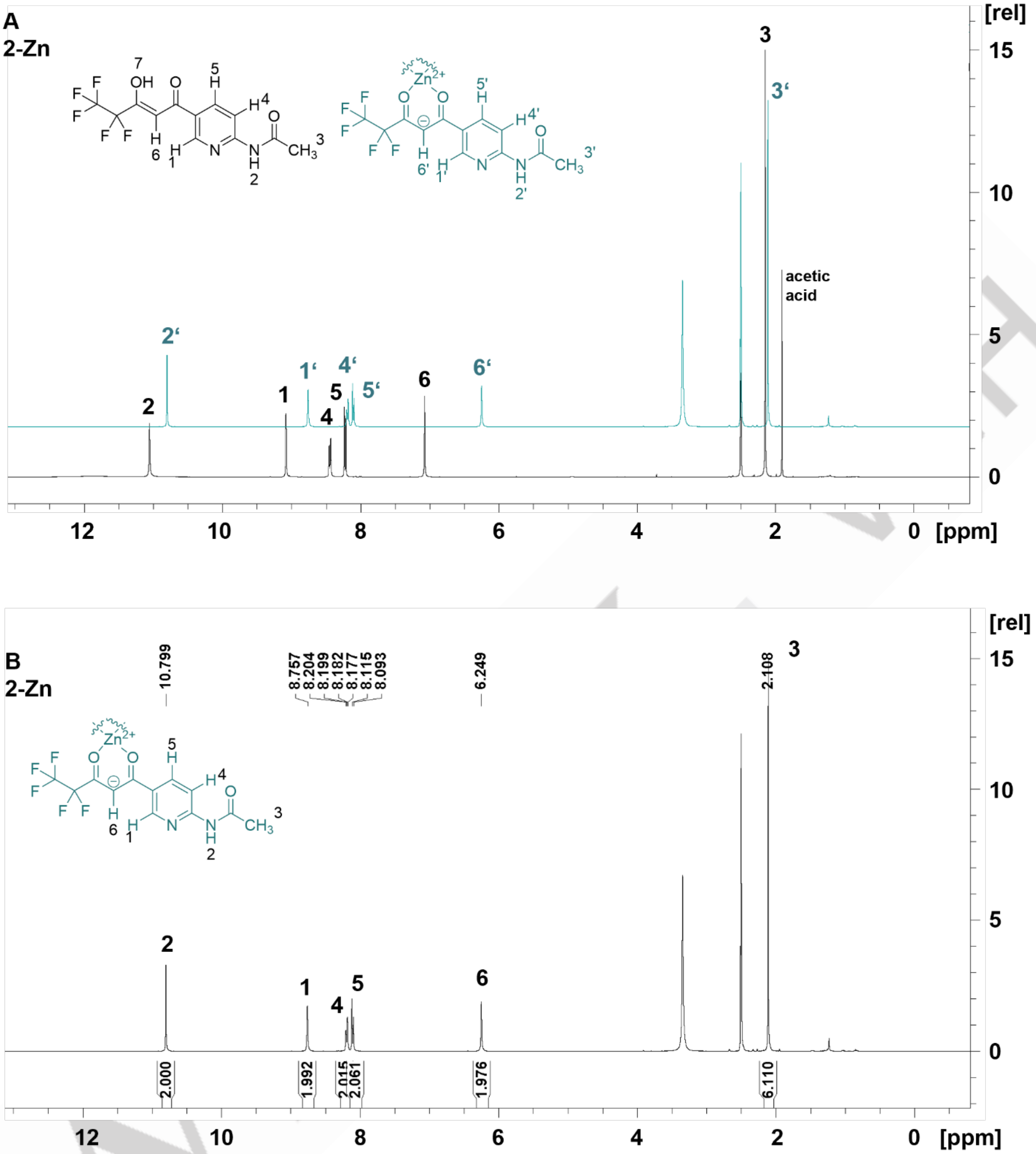

Figure S4. ^{1}H NMR spectrum of **2-Zn (A)** (Cyan) in Overlay with **1** and spectrum of **2-Zn (B)** with shift and integral annotations (400 MHz, DMSO-*d6*). Proton 7 not observed.

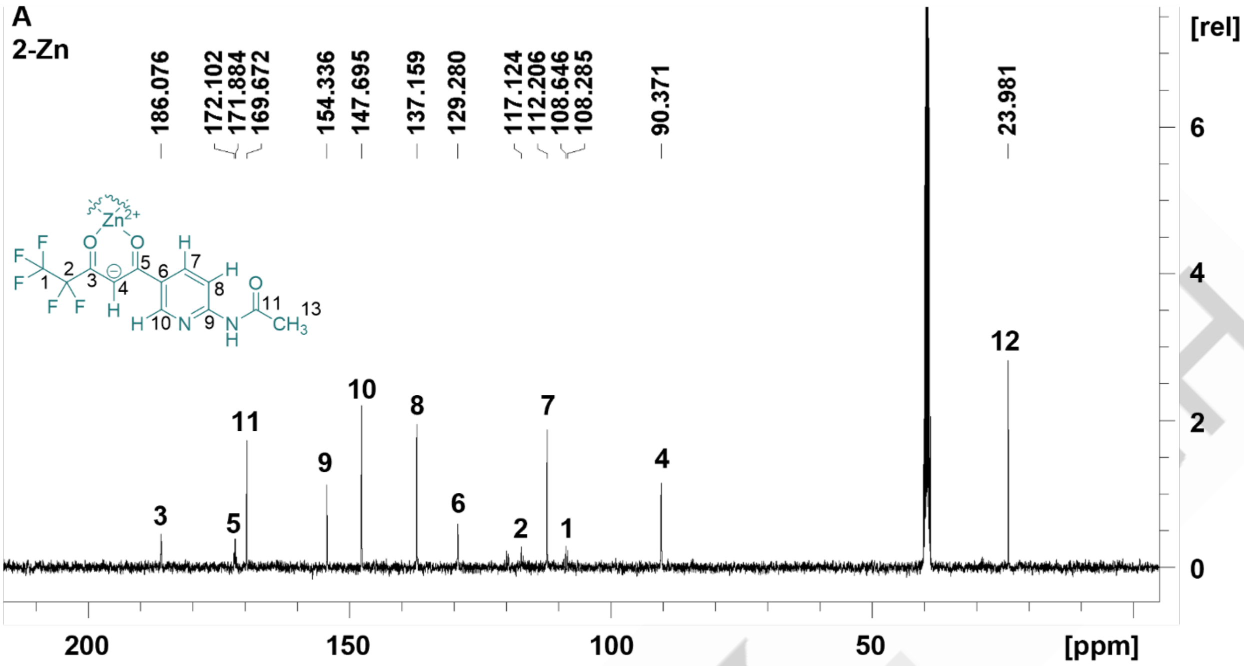

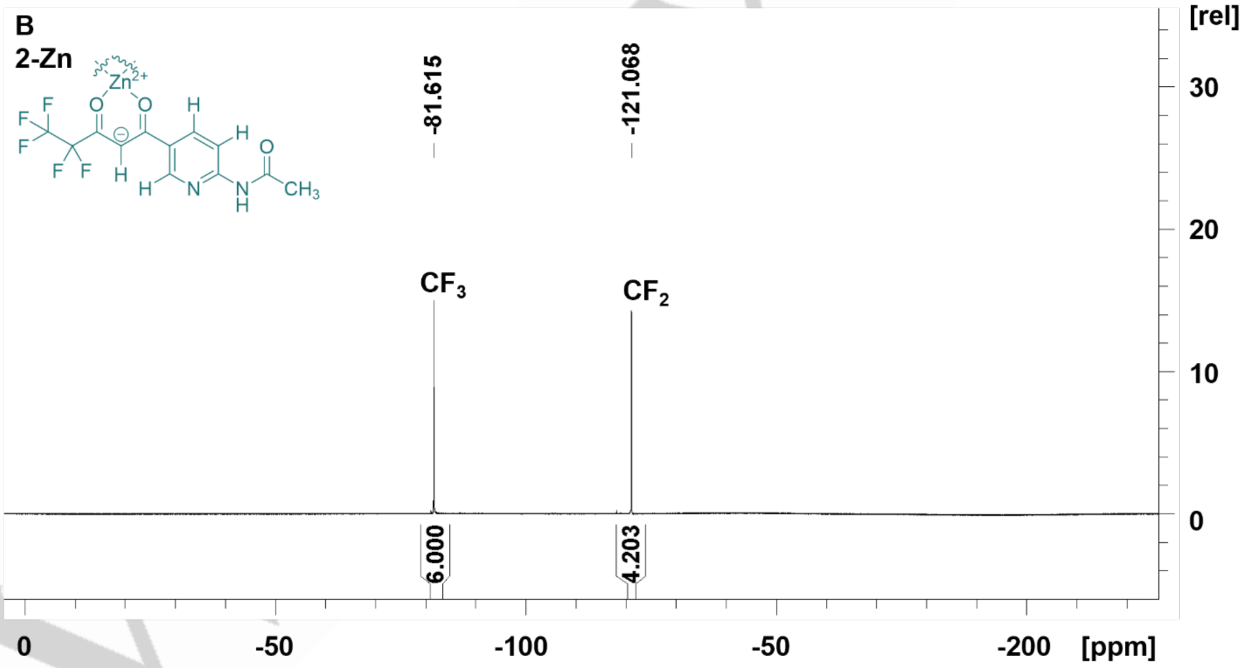

Figure S5. ^{13}C (101 MHz, DMSO-*d6*) **(A)** and ^{19}F NMR (377 MHz, DMSO-*d6*) **(B)** spectra of **2-Zn**.

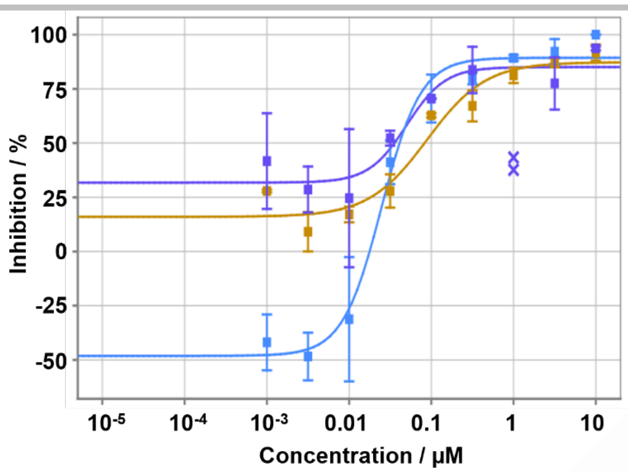

Figure S6. Inhibition of NF175 maturation for **1** in three independent runs. Each run was done in duplicates; errors denote standard deviation. Solid lines indicate curves fitted through four-parameter non-linear regression using least squares method. Purple crosses indicate outlier, removed from analysis to achieve a good curve fit.

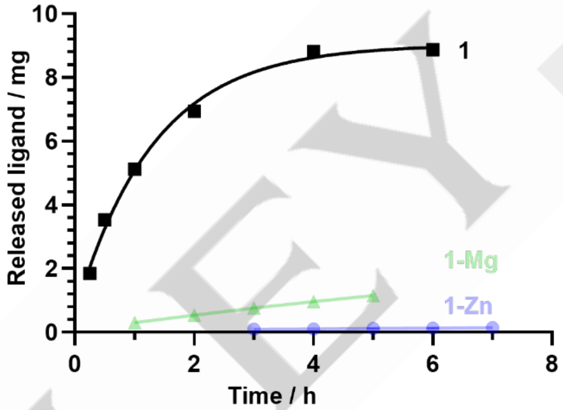

Figure S7. Release of **1** from dialysis tube to assess influence of dialysis membrane compared to release rates from **1-Mg** and **1-Zn**.

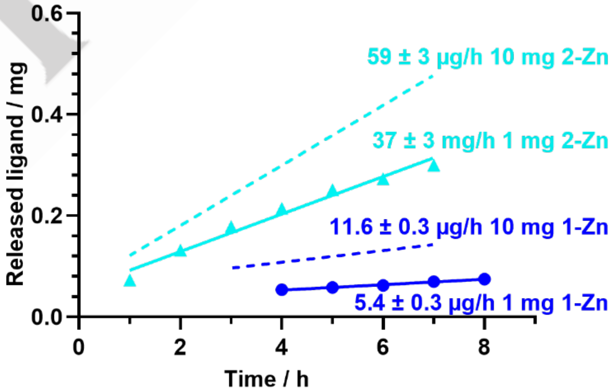

Figure S8. Assessing influence of suspended mass (10 and 1 mg) on ligand release for **1-Zn** and **2-Zn**.

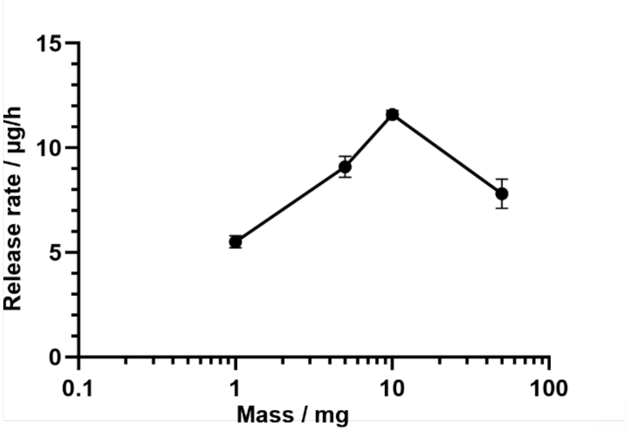

Figure S9. Assessing influence of suspended mass (1 mg, 5 mg, 10 mg, 50 mg) on ligand release rates for **1-Zn**.

WILEY-VCH

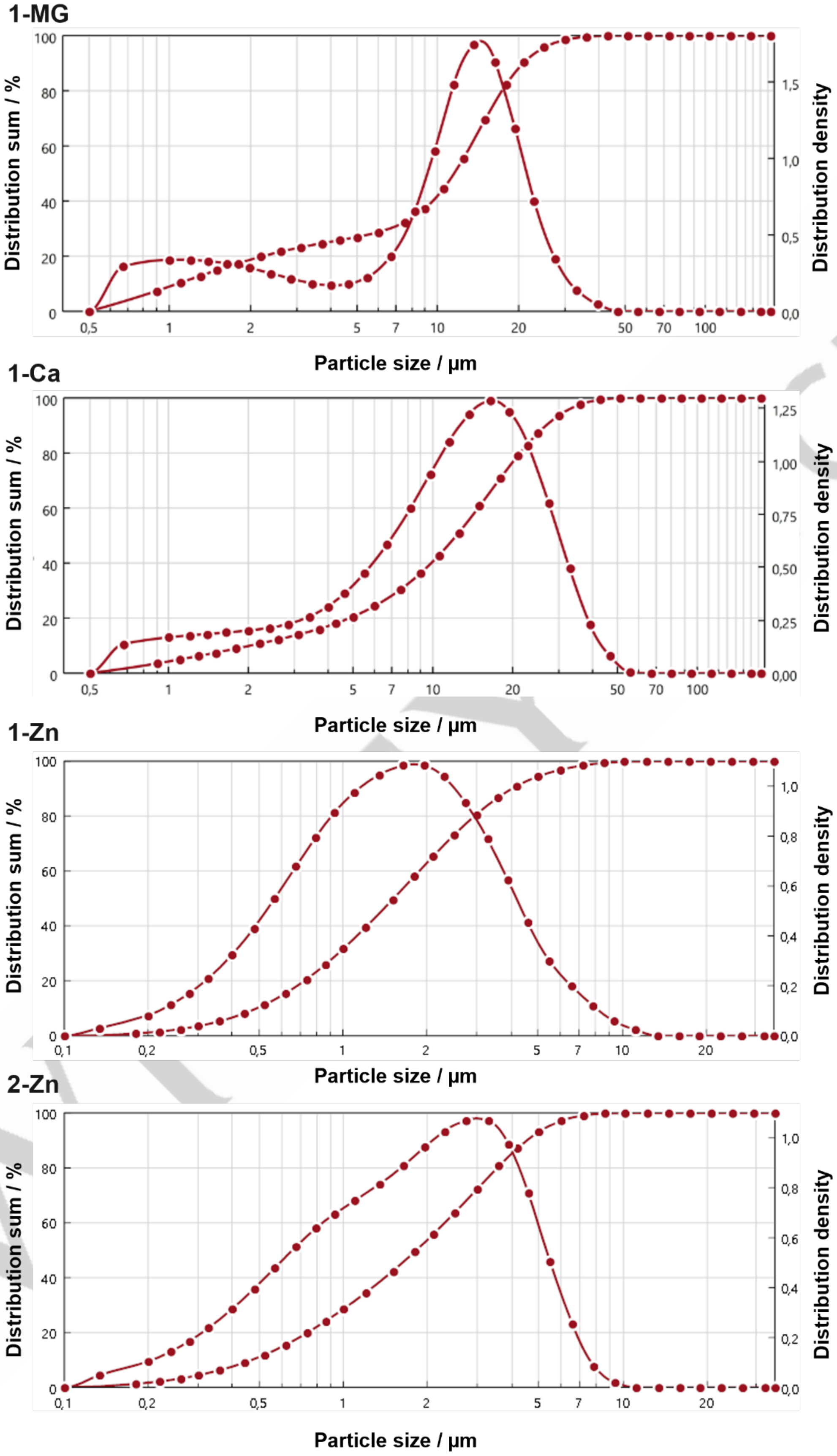

Figure S10. Particle size characterization (exemplary curves) by laser diffraction (HELOS) for metal-inhibitor complexes.
